# The American Medical Association at Portland

**Published:** 1905-08

**Authors:** 


### BUFFALO MEDICAL JOURNAL.

A Monthly Review of Medicine and Surgery.

*EDITOR:*

WILLIAM WARREN POTTER, M. D.

All communications, whether of a literary or business nature, books for review and exchanges, should be addressed to the editor 284 FRANKLIN ST., BUFFALO, N. Y.

VOL. LXI. AUGUST, 1905. NO. 1

### The American Medical Association at Portland.

REPORTS from all sources indicate that the meeting of the American Medical Association at Portland, Oregon, July 11-14, 1905, was a conspicuous success and in everything but numbers wiped out the score of all previous records. Nor was the registration small by any means; seventeen hundred and fourteen is a goodly number and far exceeds any attendance previous to five years ago.

Dr. Lewis S. McMurtry, the president, has reason to feel abundant satisfaction, not to say pride, in the record made under his administration. The orations were of an unusually high order, the section work has never been excelled,—some say never equalled,—not even in the "far East," and the business of the house of delegates in quantity and quality challenges the admiration of every person who has knowledge of such work. The social entertainments were superb.

The oration on surgery, by Dr. J. Collins Warren, of Boston, dealing with the relations of the surgeon and pathologist especially as regards benign tumors of the breast, was a masterful presentation of one of the most important surgical questions of the day and as a contribution to surgical literature will endure.

The oration on state medicine, by Dr. George Blumer, of San Francisco, dealt with the influence the acquisition of tropical territory is exercising on American medicine. It is scarcely possible to have selected a more timely subject for such a discourse and its influence is likely to be felt all over our domain. We have dealt with Dr. Stockton's oration on another page in this issue.

Dr. McMurtry's presidential address was, in the main, a review of the origin, progress, and purpose of the Association and presented certain facts in compact form which deserve to be kept in mind by every American physician. We think President McMurtry was wise in the selection of his theme, thus leaving to the orators and to the sections the discussion of the scientific problems of the period. In the course of his address the President made graceful reference to the fact that the American Medical Association had its origin in the Medical Society of the State of New York and quoted the precise language of the resolutions of that society, recommending the formation of a national organisation. Strange to say, however, the very society that gave birth to the national association is denied representation in the latter body, for technical reasons too trifling to engage the attention of full grown men. It is a question too sophomoric in character to permit of serious thought or expression.

The Association honored itself in choosing Dr. William J. Mayo, of Rochester, Minnesota, as president-elect, and the other officers also were admirably selected. Especially has the association been true to its best interests in reëlecting Dr. George H. Simmons, as secretary. The meeting in 1906 will be held at Boston.

